# Application of induced pluripotent stem cells in the conservation of endangered animals

**DOI:** 10.1186/s13287-025-04392-5

**Published:** 2025-05-28

**Authors:** Jiao Lou, Weina Li, Panlong Chen, Haiyan Chen, Amna Shakoor, Yunlong Chen, Jinlian Hua, Yan Wang, Shiqiang Zhang

**Affiliations:** 1 College of Veterinary Medicine, Shaanxi Stem Cell Engineering Research Center, Northwest A&F University, Yangling, 712100 China; 2 Key Laboratory of Livestock Biology, Northwest A&F University, Yangling, 712100 China

**Keywords:** Endangered species, Induced pluripotent stem cells, Small molecules, Primordial germ cell-like cells, Blastoids

## Abstract

The accelerating biodiversity crisis urgently demands innovative approaches that transcend traditional conservation strategies, which are often constrained by genetic bottlenecks and disease risks. Induced pluripotent stem cells (iPSCs) technology emerges as a transformative solution, enabling non-invasive genetic preservation and multi-pathway species recovery. This review synthesizes advances in reprogramming somatic cells from endangered species into iPSCs through integration-free strategies, such as mRNA, Sendai virus, episomal systems, adenoviruses and chemical induction, thereby reducing genomic instability. We highlight breakthroughs in differentiating iPSCs into functional gametes for assisted reproduction and blastoids formation for embryonic reconstruction, circumventing donor oocyte dependency and genetic homogeneity risks. Despite challenges in lineage specification and epigenetic fidelity, combining iPSC biobanking with ecosystem management enables large-scale genetic rescue. By combining these technologies with ethical frameworks and habitat restoration, the plasticity of cells may be transformed into population resilience, potentially redefining biodiversity conservation.

## Introduction

The accelerating biodiversity crisis has elevated species conservation to unprecedented global urgency [1]. Contemporary ecosystems face compounding anthropogenic pressures, with climate perturbations, habitat fragmentation, illegal wildlife trade, and emerging zoonoses collectively driving population collapses across taxonomic groups. Numerous species now confront extinction thresholds, necessitating critical evaluation of conventional preservation strategies [2]. Traditional conservation methods like habitat protection, ex situ breeding and trade regulation carry inherent risks of disease transmission and genetic bottlenecks, limiting their ability to address cascading biodiversity loss [3].

Biobanking technologies are transformative solutions to these systemic challenges, helping with genetic preservation and species recovery. Current biobanking paradigms encompass four principal cellular repositories: gametes, embryos, somatic cells and stem cells. While gamete and embryo cryopreservation maintain moderate reconstitution feasibility, their practical implementation is constrained by technical complexities in acquisition and limited post-thaw viability. Somatic cell banking presents comparative advantages in procurement simplicity and cryogenic stability, yet confronts critical barriers in de novo organism generation.

Somatic cell nuclear transfer (SCNT) technique enables somatic cell nuclei to be transferred into enucleated oocytes to produce clonal organisms [4]. However, this approach engenders substantial ethical and operational challenges. Live donor cell extraction via invasive biopsy procedures poses physiological distress and mortality risks, while post-mortem tissue collection has strict viability requirements, often damaging genetic integrity [5]. The difficulty of obtaining conspecific oocytes from endangered species limits SCNT’s use in conservation. Furthermore, SCNT-derived populations exhibit dangerous genetic uniformity, diminishing adaptive capacity against environmental stressors and pathogen evolution [6].

Recent advances in iPSCs propose a paradigm shift in species preservation strategies. iPSCs have cryopreservation resilience, and theoretically limitless expansion potential. iPSC technology offers two key conservation pathways: differentiating into functional germ cells for assisted reproduction and generating blastoids for embryo reconstruction. These methods avoid the genetic homogeneity issues of cloning and remove the need for donor oocytes.

**Fig. 1:**
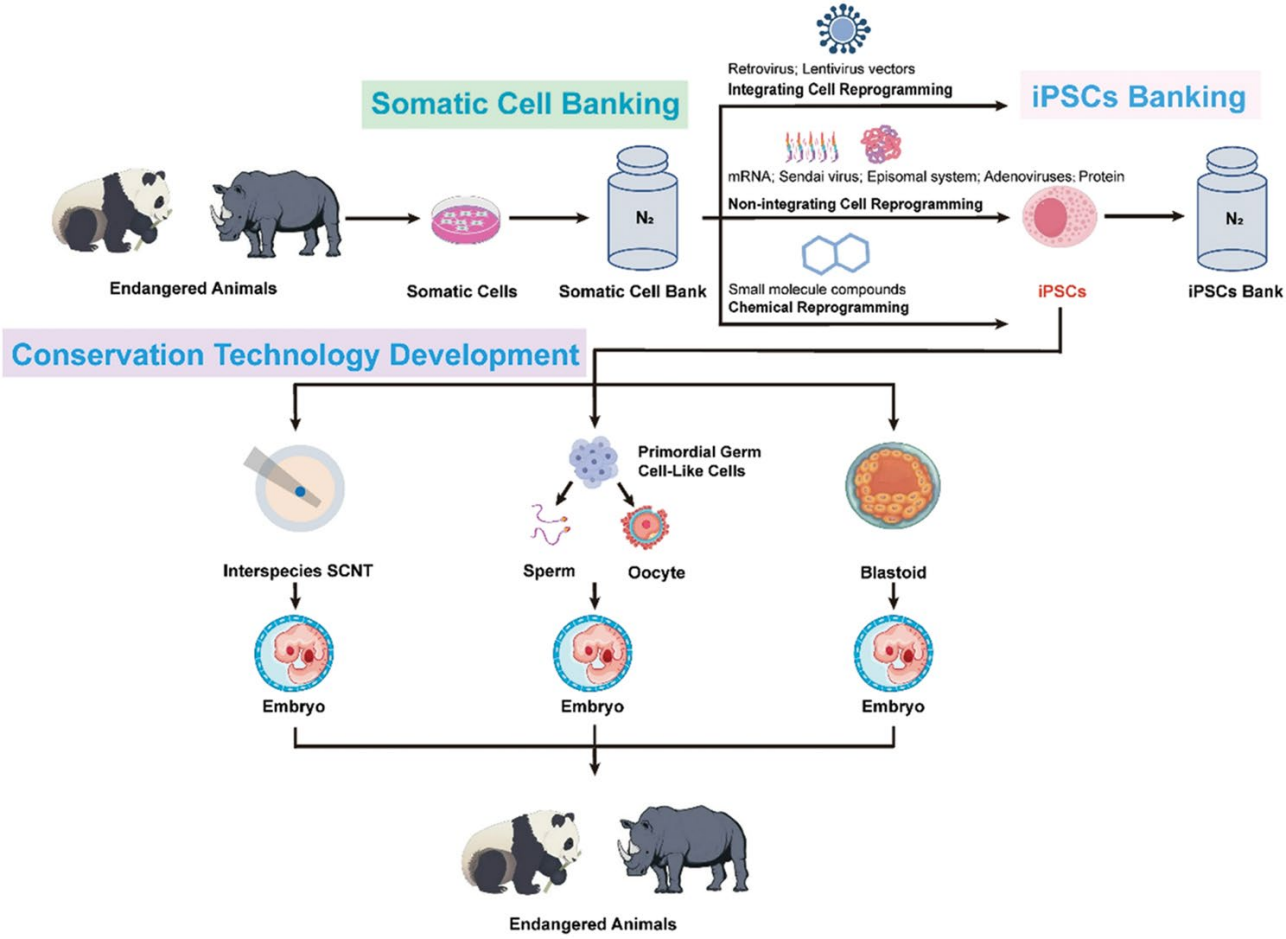
Technology roadmap for species conservation based on iPSCs. Schematic diagram of the technical pipeline for conserving endangered species using iPSCs technology. Somatic cells extracted from endangered animals are cryopreserved in a somatic cell bank and reprogrammed into iPSCs through three strategies: integrative reprogramming (retrovirus, lentivirus vectors), non-integrative reprogramming (mRNA, Sendai virus, episomal system, adenoviruses and proteins), and chemical reprogramming (small molecules). Reprogrammed iPSCs are archived in an iPSC bank. During the development of conservation technologies, iPSCs are utilized through cross-species SCNT or differentiated into functional germ cells to generate chimeric embryos. Alternatively, iPSCs may be directly differentiated into blastoids to construct functional embryos

This review summarizes current methods for generating and differentiating iPSCs, assessing how they can help save endangered species (Fig. 1). By analyzing current technological limitations and projecting future development trends, we propose a strategic framework that integrates iPSCs biobanking with ecological management to establish next-generation biodiversity preservation systems.

## iPSCs biobanking for endangered species conservation

iPSCs technology, since its emergence, has not only transformed regenerative medicine but also opened up new prospects for conserving endangered species. Cross-species cellular reprogramming enables scientists to reprogram somatic cells from endangered species to pluripotency, providing a foundation for genetic resource preservation, disease modeling, and potential population recovery. However, the application of this technology across different species still faces numerous challenges. The following sections will systematically elaborate on the research progress, current limitations, and innovative solutions of iPSCs technology in the conservation of endangered species.

### Recent progress in Establishing iPSC lines from endangered species

In 2006, Takahashi and Yamanaka generated mouse iPSCs by introducing four transcription factors: Oct4, Sox2, Klf4 and c-Myc (OSKM) [7]. Since the initial breakthrough, iPSC lines have been established for a variety of species, including endangered birds, primates, rhinoceros, and giant panda (Table 1).

**Table 1:** Establishment of iPSC lines in endangered species

Species	Reprogramming method	Characterization	Induction efficiency	References
Drill (*Mandrillus leucophaeus*)	Retroviral transduction of human OSKM in adult fibroblasts	AP activity, expression of pluripotency genes, EB formation, in vivo teratoma formation, Normal karyotype (P10)	Not mentioned	[35]
Snow leopard (*Panthera uncia*)	Retroviral transduction of human OSKM + Nanog in adult dermal fibroblasts	AP activity, expression of pluripotency genes, in vivo teratoma formation, 92% normal karyotype (P14)	0.000517%	[36]
Bengal tiger (*Panthera tigris*) Serval (*Leptailurus serval*) Jaguar (*Panthera onca*)	Retroviral transduction of human OSKM + Nanog in adult dermal fibroblasts	AP activity, expression of pluripotency genes, EB formation, in vivo teratoma formation, Normal karyotype (P14)	Bengal tiger:0.00060% Serval:0.00064% Jaguar:0.00058%	[37]
Sumatran orangutan (*Pongo abelii*)	Retroviral transduction of human OSKM in adult skin fibroblasts	AP activity, expression of pluripotency genes, EB formation, in vivo teratoma formation	Not mentioned	[38]
Ryukyu spiny rat (*Tokudaia osimensis*)	PiggyBac transposase vector transduction of mouse OSKM+/—Nanog in adult fibroblasts	Expression of pluripotency genes, in vivo teratoma formation, interspecies chimera generation with germ line contribution, 86 -91% normal karyotype(P10)	Not mentioned	[39]
Tasmanian devil (*Sarcophilus harrisii*)	Lentiviral transduction of human OSKM + Nanog + Lin28a in adult dermal fibroblasts	AP activity, expression of pluripotency genes, EB formation, in vivo teratoma formation, normal karyotype (P22)	0.0005–0.00075%	[40]
*Okinawa Rail* *(Hypotaenidia okinawae)* *Ptarmigan* *(Lagopus muta japonica)* *Blakiston’s Fish Owl* *(Bubo blakistoni)*	PiggyBac transposase vector transduction of OSKM + Nanog + Lin28a + Klf2 in adult fibroblasts	AP activity, expression of pluripotency genes, EB formation, in vivo teratoma formation, normal karyotype (P20)	Not mentioned	[41]
Sumatran rhinoceros (*Dicerorhinus sumatrensis*)	Sendai virus transduction of human OSKM in adult skin fibroblasts	Expression of pluripotency genes, EB formation, normal karyotype	Not mentioned	[42]
Bornean orangutans (*Pongo*)	Sendai virus transduction of human OSKM in peripheral blood mononuclear cells	Expression of pluripotency genes, EB formation, in vivo teratoma formation, normal karyotype (P30)	Not mentioned	[43]
Celebes Crested Macaque (*Macaca nigra*) Lar Gibbon (*Hylobates lar*) Siamang (*Symphalangus syndactylus*)	Sendai virus transduction of human OSKM in adult fibroblasts	AP activity, expression of pluripotency genes, EB formation, normal karyotype(P20)	0.12–0.14%	[44]
*Hypsugo alaschanicus* *Pipistrellus abramus*	Sendai virus transduction of human OSKM in fetal fibroblasts	AP activity, expression of pluripotency genes, EB formation	Not mentioned	[45]
Giant panda	Episomal system transduction of human OSK in adult fibroblast	AP activity, expression of pluripotency genes, EB formation, normal karyotype(P30)	0.3%	[8]

Studies on iPSCs in endangered species share common methodologies. Most rely on core transcription factor combinations like human or mouse-derived OSKM or their variants. However, species-specific adaptations are needed. These may involve adding auxiliary factors like Nanog, or using simplified combinations such as Oct4, Sox2 and Klf4 (OSK) alone, as seen in giant pandas [8]. Vector technologies have evolved from early retroviral systems to non-integrating methods, such as the Sendai virus, eliminating the risk of genomic insertion. Cell sources prioritize adult skin fibroblasts due to ethical and practical feasibility, while alternatives like peripheral blood mononuclear cells or fetal fibroblasts remain less explored. Pluripotency validation of endangered species iPSCs relies on standardized methods: alkaline phosphatase (AP) activity, pluripotency gene expression, embryoid body (EB) differentiation, and in vivo teratoma formation. Using interspecies chimeras with germline contribution in Ryukyu spiny rats for advanced functional validation confirms the naïve pluripotency of established cell lines. Karyotypic stability remains critical for long-term utility, with most iPSC lines maintaining normal karyotypes through passages. iPSCs technology has been successfully applied to establish pluripotent stem cell lines for multiple endangered species, opening new avenues for species conservation and medical research. However, its widespread application is still limited by challenges in delivery methods, differentiation efficiency, and technical hurdles in cross-species chimera research.

## Limitations of endangered species iPSCs

The process of establishing iPSC lines from endangered animals has many technical limitations. Firstly, when retroviral vectors or lentiviral vectors are used to introduce exogenous reprogramming factors into cells, these factors can integrate randomly into the host genome. This random integration may disrupt normal gene function, pose an oncogenic risk, and affect genetic stability, leading to discrepancies between the generated iPSCs and the original individual. Moreover, during the reprogramming process, endogenous pluripotency-related genes may not be fully activated or may gradually become inactive, resulting in a “partially reprogrammed” state. In this state, the iPSCs lack the desired differentiation potential and increase the risk of carcinogenesis.

To address these challenges and establish an efficient and stable iPSC system, further optimization of existing methods and techniques is essential. This requires transitioning from exogenous to endogenous gene control, reducing viral vector risks and improving iPSC quality and functionality.

### New approaches in generating iPSCs from endangered species

With the growing demand for endangered species conservation, traditional virus-based iPSC generation techniques such as retroviral or lentiviral vectors face limitations due to the risks of insertional mutagenesis. To balance efficiency and safety, researchers have developed two innovative strategies: non-integrating reprogramming through viral or plasmid delivery systems, and chemical reprogramming via small-molecules that avoids exogenous gene integration entirely. These approaches aim to minimize genomic interference while preserving cellular pluripotency, offering more controlled technical pathways for conserving genetic resources of endangered species.

#### Integration-free transgene strategies for iPSC generation

To mitigate the adverse effects of exogenous gene integration, researchers have developed several non-integrating approaches for delivering reprogramming factors. These methods include the use of RNA, Sendai virus, episomal system, adenoviruses and proteins (Table 2). These techniques aim to reduce genomic alterations and lower potential safety risks.

**Table 2:** Exogenous factor-based integration-free strategies for human iPSC generation

Species	Approaches	Reprogramming efficiency	References
Human	mRNA transduction	Fetal fibroblasts: 1.4-4% Adult fibroblasts: 0.5-2%	[10]
Human	Self-Replicative RNA transduction	Fetal Fibroblasts: 0.1-0.2% Adult fibroblasts: 0.01%	[11]
Human	MicroRNA transduction	Fibroblasts: ~10%	[12]
Human	mRNA and microRNA transduction	Fetal fibroblasts:90.7% (Single-cell reprogramming efficiency)	[13]
Human	Sendai virus transduction	Fetal fibroblasts: 0.1-1%	[14]
Human	Episomal system transduction	Fetal fibroblasts: 0.01-0.1% Adult dermal fibroblasts: <0.01%	[15]
Human	Adenoviruses transduction	Fetal fibroblasts: ~ 0.001%	[16, 46]
Human	Proteins transduction	Fibroblasts: <0.001%	[17]

By comparing various methods using cells of human, RNA technology demonstrates high reprogramming efficiency, particularly in fetal-derived fibroblasts, where its performance is most remarkable. Current RNA reprogramming approaches are primarily categorized into three types: ​chemically modified synthetic mRNA, ​self-replicating RNA, and ​microRNA [9].The hallmark advantage of these RNA technologies lies in their complete avoidance of genomic integration risks. Specifically, ​chemically modified synthetic mRNA​ achieves immunogenicity reduction through nucleotide modifications but requires frequent in vitro manipulations to sustain transcription factor expression levels [10]. In contrast, ​self-replicating RNA​ leverages the Venezuelan equine encephalitis virus replication machinery, enabling sustained OSKM factor expression for up to 10 days following a single transfection [11]. Notably, microRNAs offer distinct advantages due to their small molecular size, which enables higher transfection efficiency compared to mRNAs [12]. When co-delivered with mRNAs, microRNAs can significantly enhance reprogramming efficiency while reducing the required frequency of transfections [13]. While in efficiency Sendai virus demonstrates high reprogramming efficiency and broad applicability, necessitates caution due to potential residual viral elements and immunogenicity risks [14]. Although the episomal system sustains long-term factor expression, its efficiency plummets in adult cells, demonstrating that cellular differentiation states critically influence reprogramming difficulty [15]. In contrast, adenovirus exhibits extremely low efficacy in human cells, significantly lower than in other species, suggesting notable cross-species applicability differences [16].Protein delivery, as the safest approach without genetic material integration, is nearly impractical due to its minimal efficiency [17].

These patterns confirm the core dilemma in technology selection: high-efficiency strategies often entail operational complexity or biosafety risks, while inherently safe approaches are hindered by insufficient efficacy. Additionally, the consistent superiority of fetal cells over adult cells suggests that intrinsic cellular epigenetic characteristics may dominate reprogramming outcomes (Table 2).

Future research should focus on developing novel and highly efficient non-integrating technologies for specific applications, such as endangered species conservation, including optimized protein delivery systems to overcome dual challenges of efficiency and safety. Meanwhile, the use of small-molecule compounds to induce pluripotency without exogenous gene integration has also emerged as a key research focus.

#### Chemical reprogramming

Chemical reprogramming typically employs a phased strategy, dynamically adjusting small molecule combinations to synergistically regulate signaling pathways and epigenetic states, thereby activating the endogenous pluripotency network. The strategies for reprogramming somatic cells vary across species, with more advanced species requiring more steps. Almost all species start with core molecules like CHIR99021 and E616452 to activate growth signals and block cell specialization. Later stages add epigenetic tools like 3-Deazaneplanocin A (DZNep) and 5-azadC to remove species-specific barriers. Fish rely on AM580 in stage III, hinting their stem cells depend on retinoic acid signals to stay flexible, whereas human use JNK inhibitors and UNC0379 in stage III to address unique epigenetic locks (Table 3).

**Table 3:** Stages and progression of chemically-induced pluripotent stem cell reprogramming

Species	Donor cells	Small molecule combination	References
Mouse	fibroblasts	Stage I: VPA, CHIR99021, E-616,452, tranylcypromine (Tranyl), Forskolin Stage II: VPA, CHIR99021, E-616,452, Tranyl, Forskolin, DZNep Stage III: CHIR99021, PD0325901	[47]
Mouse	fibroblasts, embryo-derived XEN cells	Stage I: VPA, CHIR99021, E-616,452, Tranyl, Forskolin, AM580, EPZ004777 concentration change Stage II: VPA, CHIR99021, E-616,452, Tranyl, Forskolin, AM580, DZNep, 5-aza-dC, SGC0946 Stage III: CHIR99021, PD0325901	[48]
Mouse	neural stem cells, intestinal epithelial cells	Neural stem cells -CiPSCs Stage I: VPA, CHIR99021, E-616,452, Tranyl, Forskolin, Ch 55, EPZ004777 Stage II: VPA, CHIR99021, E-616,452, Tranyl, Forskolin, Ch 55, EPZ004777, DZNep Stage III: CHIR99021, PD0325901 Intestinal epithelial cells-CiPSCs Stage I: VPA, CHIR99021, E-616,452, Tranyl, Forskolin, AM580 Stage II: VPA, CHIR99021, E-616,452, Tranyl, Forskolin, Ch 55, DZNep Stage III: CHIR99021, PD0325901	[49]
Mouse	fibroblasts, neural stem cells, hepatocytes	Fibroblasts, Neural stem cells -CiPSCs Stage I: CHIR99021, Brdu, E-616,452, Forskolin, VPA, AM580, EPZ5676, DZNep, SGC0946 Stage II: CHIR99021, PD0325901 Hepatocytes -CiPSCs Stage I: CHIR99021, Brdu, E-616,452, Forskolin, VPA, AM580, EPZ5676, DZNep, SGC0946 Stage II: HCM medium (Lonza) Stage III: CHIR99021, PD0325901	[50]
Mouse	spermatogonial stem cells	Stage I: SGC707, Vitamin C, Epigallocatechin Gallate, Daphnetin, Tauroursodeoxycholic acid Stage II: CHIR99021, PD0325901, LIF (2iL-medium)	[51]
Fish (Koi)	fibroblasts	Stage I: VPA, CHIR99021, E-616,452, Tranyl, Forskolin, DZNep, EPZ004777 concentration change Stage II: VPA, CHIR99021, E-616,452, Tranyl, Forskolin, DZNep, AM580, SGC0946, 5-aza-dC Stage III: PD0325901, A8301, CHIR99021	[52]
Human	fibroblasts, adipose-derived mesenchymal stromal cells	Stage I: CHIR99021, E-616,452, TTNPB, Y27632, SAG, ABT-869 Stage II: CHIR99021, E-616,452, TTNPB, Y27632, SAG, ABT-869, Tranyl, 5-Aacytidine, JNKIN8 Stage III: CHIR99021, E-616,452, Y27632, PD0325901, Tranyl, VPA, EPZ004777, DZNep, UNC0379 Stage IV: CHIR99021, PD0325901, SB590885, IWP-2, Y27632	[53]
Human	fibroblasts, adipose-derived mesenchymal stromal cells	Stage I: CHIR99021, E-616,452, TTNPB, SAG, EPZ5676, DZNep, Ruxolitinib, VTP50469, AKT Kinase Inhibitor, JNKIN8, SETD2-IN-1 Stage II: CHIR99021, E-616,452, TTNPB, SAG, EPZ5676, DZNep, Ruxolitinib, BIRB796, SGC-CBP30, Dorsormorphin, VTP50469, 5-Iodotubercidin, 5-Azacytidine, AKT Kinase Inhibitor, CX-4945 Stage III: CHIR99021, Y27632, PD0325901, SB590885, Tranyl, VPA, DZNep, EPZ5676	[54]

The use of small molecule compounds to induce iPSCs offers multiple advantages. These include enhanced safety by avoiding viral vectors or transgenic factors, thereby reducing the risk of potential genomic insertional mutations. The process is also more convenient, as small molecule compounds can be directly added to the culture medium, and it is cost-effective, being less expensive than complex viral transduction systems or gene-editing technologies. Additionally, adjusting the concentration of small molecule compounds and the treatment duration allows for better control over the cell fate conversion process. Given these advantages, small molecule-induced iPSCs technology holds significant promise for the conservation of endangered species.

However, achieving chemical reprogramming in endangered species still faces challenges. For instance, the reprogramming efficiency of small molecule-induced iPSCs (approximately 0.1%) is lower than methods using Sendai virus or mRNA, and the underlying molecular mechanisms remain complex, likely involving multiple signaling pathways that are not yet fully understood. Furthermore, identifying the optimal combinations of small molecule compounds for endangered species requires extensive screening and optimization. Despite these challenges, chemical reprogramming provides new possibilities for preserving the genetic information of endangered species.

## Potential pathways for restoring endangered species with iPSCs

The restoration of endangered animal individuals using iPSCs can be potentially achieved through two primary pathways (Fig. 1). One pathway involves differentiating iPSCs derived from the endangered species into primordial germ cells (PGCs), which can then mature into functional sperm or oocytes. These gametes can be used for in vitro fertilization (IVF) to form early-stage embryos, which are then transferred to a surrogate mother’s uterus for full development.

Alternatively, iPSCs can be directly induced under specific culture conditions to form “blastoids” structures that resemble blastocysts at an early stage of natural embryonic development. If the blastoids successfully develop to a certain stage in vitro, they can be transferred into the uterus of a surrogate mother, with the aim of completing the entire gestation period and eventually giving birth to offspring.

### In vitro gamete production for conservation

Early studies have demonstrated that iPSCs have the ability to differentiate into primordial germ cell-like cells (PGCLCs) under in vitro conditions and further develop into functional gametes. In endangered species, PGCLCs have been robustly induced through a two-step protocol from embryonic stem cells (ESCs) derived from the Southern white rhinoceros and iPSCs derived from the Northern white rhinoceros, but the generation of functional germ cells has not yet been achieved [18].

In mouse models, researchers have successfully differentiated iPSCs into functional oocytes or sperm, which are capable of producing healthy offspring. For example, the work by Hayashi et al. has shown the potential to generate functional gametes from mouse pluripotent stem cells, opening new prospects for reproductive medicine. In this mouse experiments, female ESCs and iPSCs were induced into PGCLCs, which underwent proper development in reconstituted ovaries in vitro and matured further into fully functional oocytes upon transplantation in vivo [19]. And the work by Zhou et al. has demonstrated that mouse ESC-derived PGCLCs entered meiosis in vitro, undergoing key processes of in vivo meiosis, including chromosomal synapsis and recombination, and finally differentiated into haploid spermatid-like cells. These spermatid-like cells successfully fertilized oocytes by intracytoplasmic sperm injection, and the resulting embryos underwent embryonic development, resulting in fertile offspring that gave birth to the next generation [20].

These studies have paved the way for developing methods to differentiate germline precursor cells from PSCs of different species. Although each species has its unique characteristics, many cellular mechanisms appear to be conserved (Table 4). Recent progress has been made in differentiating PGCLCs from PSCs in non-rodent species such as rabbits, pigs, and cattle [21–23]. However, despite the successful induction of PGCLCs fate as evidenced by the expression of germ cell markers at the mRNA and protein levels, no reports have yet demonstrated the functionality of these PGCLCs in any non-rodent species.

**Table 4:** Induction of PGCLCs in different species

Species	Induction steps		Cytokines and duration	Characterization	Efficiency	References
Mouse	Epiblast-PGCLCs		BMP4, LIF, SCF, BMP8b, EGF (84–132 h)	Marker Expression, Gene Expression Analysis, Cell Differentiation and Integration, Functional Maturation	100%	[55]
Mouse	ESCs/iPSCs-EpiLCs-PGCLCs		I: Activin A, bFGF (2 Day) II:BMP4, LIF, SCF, BMP8b, EGF (3 Day)	Marker Expression, Gene Expression Analysis, Cell Differentiation and Integration, Functional Maturation	12%	[56]
Mouse	ESCs/iPSCs-EpiLCs-PGCLCs		I: Activin A, bFGF (2 Day) II:BMP4, LIF, SCF, BMP8b, EGF (3 Day)	Marker Expression, Gene Expression Analysis, Cell Differentiation and Integration, Functional Maturation	Not mentioned	[19]
Human	ESCs/iPSCs- Mesoderm like cells -PGCLCs		I: Activin A, bFGF, Y27632 (2 Day) II:BMP4, LIF, Y27632	Marker Expression, Gene Expression Analysis, Cell Differentiation and Integration, Functional Maturation	20%	[57]
Mouse	ESCs-EpiLCs-PGCLCs		I: Activin A, bFGF (2 Day) II:BMP4, LIF, SCF, BMP8α, EGF (6 Day)	Marker Expression, Gene Expression Analysis, Cell Differentiation and Integration, Functional Maturation	11.2%	[20]
Porcine	iPSCs-EpiLCs-PGCLCs		I: Activin A, bFGF (2 Day) II:BMP4, LIF, SCF, BMP8b, EGF (3 Day)	Marker Expression, Gene Expression Analysis, Cell Differentiation and Integration, Functional Maturation	Not mentioned	[58]
Porcine		EPSCs-Pre-differentiation-PGCLCs	I: Activin A, Y27632 (2 Day) II:BMP2, LIF, SCF, EGF, Y27632 (3 Day)	Marker Expression, Gene Expression Analysis	11.1%	[21]
Rabbit		PSCs-PGCLCs	BMP4, LIF, SCF, EGF, Y27632	Marker Expression, Gene Expression Analysis	20–50%	[22]
Rat		ESCs-EpiLCs-PGCLCs	I: Activin A, bFGF (48–72 h) II:BMP4, LIF, SCF, EGF (3 Day)	Marker Expression, Gene Expression Analysis, Cell Differentiation and Integration, Functional Maturation	Not mentioned	[59]
White rhinoceros		ESCs/iPSCs-Preinduction-PGCLCs	I: BMP4, CHIR99021, Y27632 (1 Day) **II**:BMP4, LIF, SCF, EGF , Y27632 (4 Day)	Marker Expression, Gene Expression Analysis, Cell Differentiation and Integration	Not mentioned	[18]
Marmoset		iPSCs-PGCLCs	BMP4, LIF, EGF, SCF, Y27632 (8 Day)	Marker Expression, Gene Expression Analysis, Cell Differentiation and Integration	15-40%	[60]
Cattle		ESCs-Pre-differentiation - PGCLCs	I: BMP4, CHIR99021, IWR1, Y27632 (1 Day) **II**:BMP4, LIF, SCF, EGF, CHIR99021, IWR1, Y27632 (4 Day)	Marker Expression, Gene Expression Analysis, Cell Differentiation and Integration	10–20%	[23]

Generating gametes from iPSCs advances both reproductive biology research and endangered species conservation. For endangered animals whose populations have drastically declined due to habitat loss, environmental changes, or diseases, the technology of differentiating iPSCs into sperm or oocytes could provide a lifeline for the continuation of these species. By using these in vitro-generated gametes in assisted reproductive technologies, such as IVF, scientists may be able to increase the number of endangered animals and help to restore their genetic diversity. However, this technological pathway faces numerous challenges, including technical complexity and low differentiation efficiency (Table 4).

### Blastoids production for conservation

**Fig. 2:**
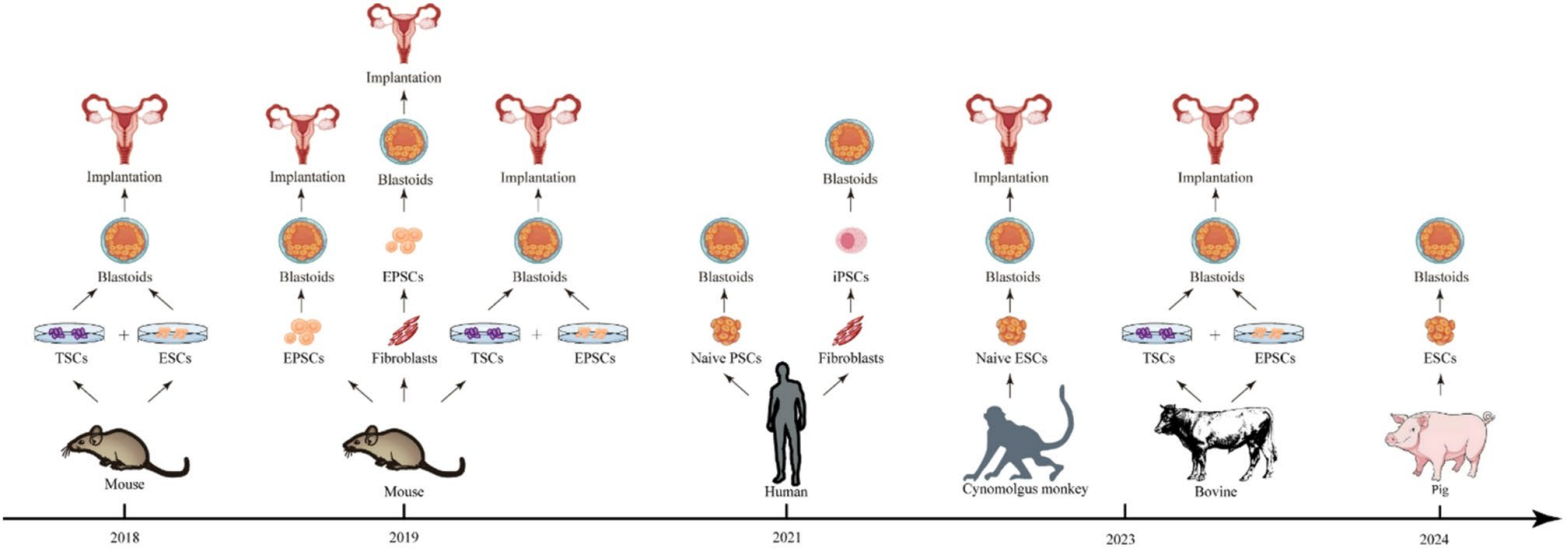
Successful development of blastoids in different species. Milestone achievements in the PSCs differentiation into blastoids. Through a timeline spanning from 2018 to 2024, the research breakthroughs extend from mouse and human to cynomolgus monkey, bovine, and pig

The development of blastoid technology provides a novel platform for the conservation of endangered species. The key point of this technology is the use of stem cells to reconstruct the key structure of early embryos, known as the blastocysts, either through spontaneous differentiation or the assembly of different cell types (Fig. 2). This process not only aids in understanding the mechanisms of early animal development but also opens new avenues for assisted reproductive technique and the preservation of genetic resources.

Blastoid formation through in vitro spontaneous differentiation has been reported in mouse expanded pluripotent stem cells (EPSCs) [24], human naïve PSCs [25–27], as well as cynomolgus monkey and porcine ESCs [28, 29](Fig. 2). In the case of human, two methods have been developed to induce the spontaneous differentiation of human naïve PSCs into blastoid structures: the hypoblast differentiation followed by trophectoderm differentiation (HT) method and the trophectoderm differentiation followed by hypoblast differentiation (TH) method. In the HT method, the culture medium for PSCs is changed to Hypoblast Differentiation Medium (HDM) starting on day 1, and then further changed to Trophectoderm Differentiation Medium (TDM) on day 3, with TDM being replaced every two days until blastoid structures are observed around days 6 to 8. In the TH method, cells are first treated with TDM until the appearance of cavity structures (usually taking about 6 to 7 days), followed by a switch to HDM for an additional 1 to 2 days of culture. Both methods successfully promote the self-organization of PSCs into blastoid structures with inner cell mass and trophoblast-like cell characteristics, with the HT method showing greater robustness [30].Surprisingly, in the same year, Jose M. Polo and his colleagues directly reprogrammed fibroblasts into blastoids, establishing a human blastocyst model [31]. The process of generating Blastoids involves a two-step reprogramming approach. The first step was to convert fibroblasts into iPSCs, a process that typically takes about three weeks. During this phase, cells gradually lose their fibroblast characteristics and acquire the features of PSCs through the guidance of a series of factors. The second step involves further differentiating these iPSCs into Blastoids. This process requires specific media composed of DMEM/F-12, Neurobasal, B27, and N2 supplements, along with growth factors and small molecules such as CHIR99021, Bone Morphogenetic Protein 4 (BMP4), Epidermal Growth Factor​(EGF), A83-01, SB431542, and Valproic Acid (VPA), combined with 3D aggregation and low-oxygen culture to drive fibroblast reprogramming and self-organization into blastocyst-like structures. The resulting iBlastoids generally exhibit characteristics similar to early embryos, including pluripotency and differentiation potential [31]. This provides a new approach for the restoration of endangered species.

In addition to spontaneous differentiation, there are also reports of assembling EPSCs and trophoblast stem cells (TSCs) to form blastoids in mouse and bovine [32, 33]. Taking the bovine experimental model as an example, researchers first maintained bovine EPSCs in an expanded pluripotent state with bidirectional differentiation potential through a combination of small molecule compounds and growth factors. Subsequently, these EPSCs were co-cultured with TSCs at a specific ratio in a 3D microenvironment, utilizing microfluidic technology to simulate the mechanical stress stimuli within the oviduct. The results demonstrated that the two cell types self-organized to form blastoid structures with a diameter of approximately 120–150 μm. These structures exhibited transcriptomic profiles highly similar to those of naturally fertilized bovine blastocysts and were capable of sustaining development in vitro for up to 7 days.

Although the generation of blastoids has been successfully achieved in some species, the application of blastoid technology still faces many challenges. Firstly, the formation of blastoids relies on complex culture conditions and precisely regulated signaling pathways. For non-model organisms, especially endangered species, the relevant biological knowledge and technical methods are still in the early stages of exploration, leading to significant uncertainties. Furthermore, even if blastoids are successfully generated, it remains a substantial technical challenge to ensure their stable development into complete embryos, successful implantation, and maturation within a surrogate mother. Additionally, potential issues such as genetic stability, epigenetic changes, and safety and ethical concerns associated with blastoid technology cannot be overlooked. Moreover, the use of this technology for the restoration of endangered species must be carefully considered in terms of its potential impact on ecosystems.

To address the key technical bottlenecks in blastoids cultivation and development, single-cell transcriptome sequencing can be employed to decipher the spatiotemporal activation patterns of critical signaling pathways, such as Wnt/Bone Morphogenetic Protein, during embryonic development in various endangered species. This approach enables the design of species-specific induction media components, thereby providing a robust strategy to enhance the developmental integrity of blastoids.

## Future prospects

The future of protecting endangered species through iPSCs technology lies in integrating cutting-edge biotechnological innovations with ecosystem-scale management, thereby constructing a multidimensional strategy that transcends traditional conservation models. This strategy leverages next-generation iPSC systems developed through the combination of small molecule epigenetic modulators and clustered regularly interspaced short palindromic repeats (CRISPR)-optimized gene regulatory circuits [34]. These systems enable precise cellular reprogramming to overcome existing challenges such as low reprogramming efficiency and genomic instability, while establishing a pluripotent cell bank with preserved genetic diversity. At the technical implementation level, single-cell sequencing technologies, transcriptomic analysis, and CRISPR/Cas9 platforms provide a solid foundation for cross-species PGCLCs differentiation and blastoids development. iPSC-driven species conservation offers solutions for protecting endangered life, enabling dynamic biodiversity conservation and ultimately reconstructing ecological balance.

## Data Availability

No data was used for the research described in the article.

## Abbreviations

iPSCs: Induced pluripotent stem cells
SCNT: Somatic cell nuclear transfer
AP: Alkaline phosphatase
EB: Embryoid body
OSKM: Oct4, Sox2, Klf4, c-Myc
OSK: Oct4, Sox2, Klf4
VPA: Valproic acid
DZNep: 3-Deazaneplanocin A
Tranyl: Tranylcypromine
CiPSCs: Chemical induced pluripotent stem cells
ESCs: Embryonic stem cells
PGCs: Primordial germ cells
IVF: In vitro fertilization
PGCLCs: Primordial germ cell-like cells
EPSCs: Expanded pluripotent stem cells
BMP4: Bone morphogenetic protein 4
EGF: Epidermal Growth Factor
TSCs: Trophoblast stem cells
PSCs: Pluripotent stem cells
HT: Hypoblast differentiation followed by trophectoderm differentiation
TH: Trophectoderm differentiation followed by hypoblast differentiation
HDM: Hypoblast differentiation medium
TDM: Trophectoderm differentiation medium
CRISPR: Clustered regularly interspaced short palindromic repeats
